# Effect of Vanadium Addition on the Wear Resistance of Al_0.7_CoCrFeNi High-Entropy Alloy

**DOI:** 10.3390/ma17236021

**Published:** 2024-12-09

**Authors:** Marzena Tokarewicz, Malgorzata Gradzka-Dahlke, Wojciech J. Nowak, Andrzej Gradzik, Miroslaw Szala, Mariusz Walczak

**Affiliations:** 1 Faculty of Mechanical Engineering, Bialystok University of Technology, Wiejska 45 C, 15-351 Bialystok, Poland; marzena.tokarewicz@sd.pb.edu.pl; 2 Faculty of Mechanical Engineering and Aeronautics, Rzeszow University of Technology, Powstanców Warszawy 12, 35-959 Rzeszow, Poland; w.nowak@prz.edu.pl (W.J.N.);; 3 Department of Materials Engineering, Mechanical Engineering Faculty, Lublin University of Technology, Nadbystrzycka 36, 20-618 Lublin, Poland; m.szala@pollub.pl (M.S.); m.walczak@pollub.pl (M.W.)

**Keywords:** high-entropy alloys, wear resistance, Al_0.7_CoCrFeNi, Al_0.7_CoCrFeNiV_0.5_

## Abstract

High-entropy alloys are of interest to many researchers due to the possibility of shaping their functional properties by, among other things, the use of alloying additives. One approach to improving the wear resistance of the AlCoCrFeNi alloy is modification through the addition of titanium. However, in this study, an alternative solution was explored by adding vanadium, which has a completely different effect on the material’s structure compared to titanium. The effect of vanadium additives on changes in the microstructure, hardness, and wear resistance of the Al_0.7_CoCrFeNi alloy. The base alloys Al_0.7_CoCrFeNi and Al_0.7_CoCrFeNiV_0.5_ were obtained by induction melting. The results showed that the presence of vanadium changes the microstructure of the material. In the case of the base alloy, the structure is biphasic with a visible segregation of alloying elements between phases. In contrast, the Al_0.7_CoCrFeNiV_0.5_ alloy has a homogeneous solid solution bcc structure. The presence of vanadium increased hardness by 33%, while it significantly reduced friction wear by 73%. Microscopic observations of friction marks indicate differences in the wear mechanisms of the two materials.

## 1. Introduction

In recent years, high entropy alloys (HEAs) have attracted significant interest due to their good properties and interesting structural features [1,2,3]. Traditional alloys are characterized by the fact that they consist of one or two main elements and contain alloying additives in small amounts. HEAs are distinguished by the fact that they are made of five or more principal block elements between five and thirty-five atomic percentages. This compositional approach provides the potential for obtaining an unlimited number of alloy combinations with different properties. High-entropy alloys may prove to be the solution to the challenges that evolving technology poses to materials. Researchers are investigating these innovative materials because of their broad potential for use in medicine [4,5,6], aerospace industry [7,8], as well as other demanding areas [9,10,11].

Combining different elements creates virtually unlimited possibilities for obtaining materials with the desired functional properties [12]. One group of applications where HEA alloys may prove outstanding is friction systems for machinery and equipment. In many fields of technology, wear-resistant materials are being sought and developed. Researchers are investigating the tribological properties of high-entropy alloys in different environments and using different methods. The tests have been carried out in dry conditions or using lubricant and also at room or elevated temperatures [13,14,15,16,17].

The AlCoCrFeNi group of HEAs are preeminently multi-master alloy series. Researchers study their mechanical properties as well as their functional properties. Investigations into the influence of the content of individual components (mainly aluminum) on their properties are also very common. With increasing aluminum content in the Al_x_CoCrFeNi alloy, the fcc phase changes to the bcc phase [18,19,20]. For the x in molar ratio, the structure changes as follows: fcc is present when x ≤ 0.4, a mixed fcc and bcc phase appears between 0.5 ≤ x ≤ 0.9, a single bcc phase is present when x ≥ 0.9 [21]. An increase in aluminum content also results in an increase in hardness and a decrease in the ductility of the alloy. The authors’ previous research has demonstrated that the Al_0.7_CoCrFeNi alloy offers a good balance between hardness and ductility [22], although its tribological properties are less favorable [23]. Researchers are also investigating the effect of the addition of other elements on the properties of this Al_x_CoCrFeNi alloy. Xu et al. [24] investigated the effect of titanium addition on the microstructure and tribological properties of AlCoCrFeNi alloys. The positive effect of titanium addition on the wear properties of the Al_x_CoCrFeNi alloy is also confirmed by other studies [25,26,27]. The titanium content significantly alters the structure of the AlCoCrFeNiTi_0.5_ alloy. It leads to the formation of a honeycomb-shaped interdendrite (ID) along with the intertwined structure in the region of dendrite. Titanium in these alloys typically appears in the form of fine precipitates of hard phases. This changes the wear mechanism. Layers containing oxide wear products are formed on the friction surface to protect against wear [24,25]. In addition, the AlCoCrFeNiTi_0.5_ alloy has a high degree of brittleness. In the case of materials obtained by induction melting, internal stresses cause cracking even during slow cooling in the furnace [23]. Therefore, exploring alternative modifications to the Al alloy that could enhance its wear resistance appears to be an interesting and valuable research direction.

Another solution could be solution hardening of the alloy, e.g., by the addition of vanadium. Dong et al. [28] studied the effect of vanadium addition on the properties of AlCoCrFeNi alloys. The vanadium content in the investigated alloys varied from 0 to 1 in molar ratio. The addition of vanadium decreased the elemental segregation. In addition, it caused a lattice distortion and consequently increased the lattice constant. The researchers indicated that the reason for this is that vanadium has the second largest atomic radius of all the components in the alloy. The highest plastic strain and compressive strength were noted in the AlCoCrFeNiV_0.2_ alloy. Meanwhile, the AlCoCrFeNiV alloy exhibited the highest yield strength and hardness.

The aim of this study was to analyze the effect of a 0.5 molar vanadium addition on the change in the wear mechanism and the extent of wear in the Al_0.7_CoCrFeNi alloy.

## 2. Materials and Methods

High-entropy alloys Al_0.7_CoCrFeNi and Al_0.7_CoCrFeNiV_0.5_ were obtained by induction melting in a protective argon atmosphere. The purity of the raw materials was above 99.9%. The samples were re-melted three times to achieve chemical homogeneity. Samples prepared for microstructure investigation were ground, polished with a diamond suspension, and etched with aqua regia.

Crystal structures of manufactured alloys were analyzed by XRD using a Bruker D8Advance diffractometer, Billerica, USA with a Cu Kα source. A high-resolution scanning electron microscope (SEM-FIB DualBeam Scios 2, Thermo Scientific, Waltham, MA, USA) was used to characterize the microstructure, chemical composition, and friction paths. An Energy Dispersive Spectroscopy (EDS) detector was used to analyze the chemical composition of the sample. Hardness was measured by the Vickers method under a load of 10 kG using an INNOVATEST hardness tester, Maastricht, Netherlands. For friction tests, specimens with a diameter of 32 mm and a thickness of 6 mm were cut with a wire-cutting machine.

The friction tests were performed in ball-on-disc mode by the Bruker UMT-2 tribometer, Billerica, USA. The applied load was 10 N, the sliding speed was 0.1 m/s, and the total test time was 1800 s. The counter sample was made of Al_2_O_3_. A ball with a diameter of 6 mm was used as friction counterpart. The wear was measured using a linear method on the friction trace as groove depth using a Keyence VHX 7000 digital microscope, Mechelen, Belgium, equipped with a laser surface scanning function and the ability to analyze surface topography in all directions, including the radial direction.

Observations of the friction tracks were also carried out to analyze differences in wear mechanisms. These investigations were carried out using a Keyence VHX 7000 digital microscope, Mechelen, Belgium and a SCIOS 2 scanning electron microscope, Thermo Fisher Scientific, Waltham, USA. In addition, a focused ion beam (FIB) electron microscope was used to perform cross sections on the friction tracks to compare changes in the surface layer after friction tests on the materials studied.

## 3. Results and Discussion

### 3.1. Microstructure

**Figure 1:**
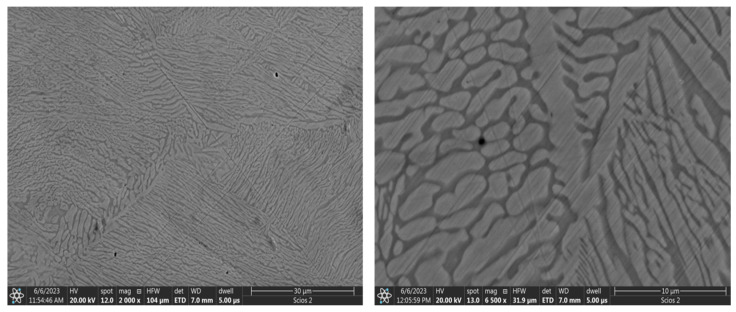
SEM image of the Al_0.7_CoCrFeNi alloy microstructure.

**Figure 2:**
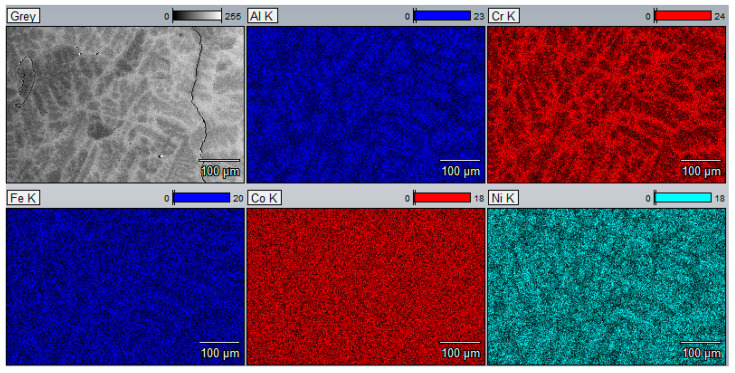
EDS maps of the Al_0.7_CoCrFeNi alloy.

The structure of the base Al_0.7_CoCrFeNi alloy after casting is two phased, consisting of light dendrites and dark interdendritic spaces (Figure 1). This corresponds to the quantitative content of the fcc and bcc phases, respectively. An analogous two-phase structure for alloys of the Al_x_CoCrFeNi series with similar aluminum content can also be found in publications [18,19,21,22]. Note the differentiation in the chemical composition of the individual components of the structure. The dendritic phase is rich in aluminum and nickel, while the interdendritic spaces show a higher chromium and iron content. Cobalt is evenly distributed in both phases (Figure 2).

**Figure 3:**
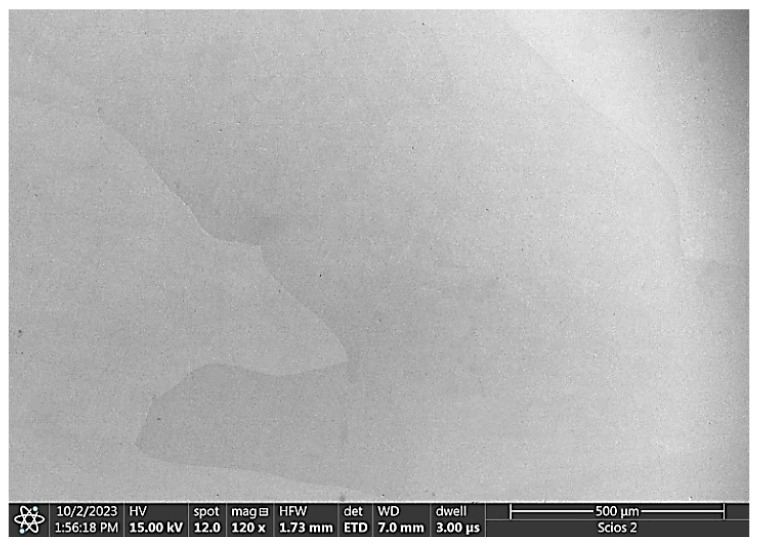
SEM image of the Al_0.7_CoCrFeNiV_0.5_ alloy microstructure.

The 0.5 molar vanadium addition resulted in the homogenization of the material. Only large homogeneous grains of the solid solution of the alloying elements are visible in the microstructure images (Figure 3). Such an effect of vanadium on the structure homogenization of the AlCoCrFeNi alloy has also been noted by other researchers [28].

**Figure 4:**
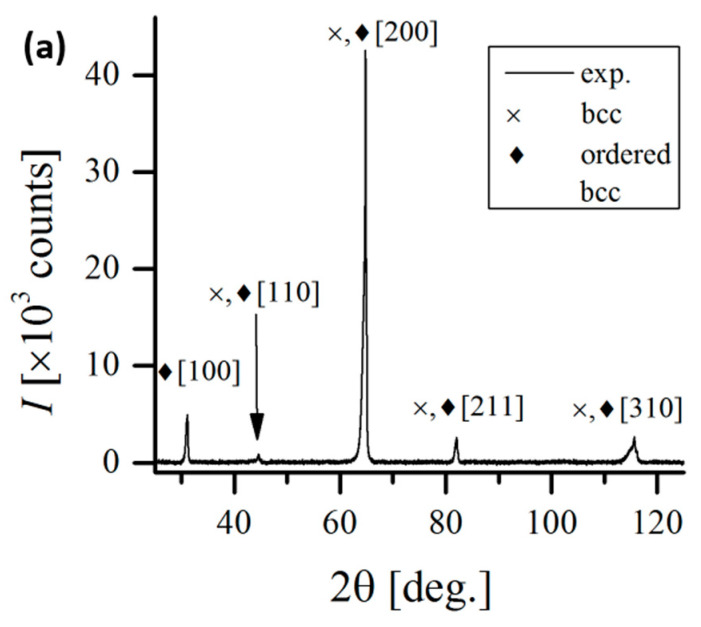

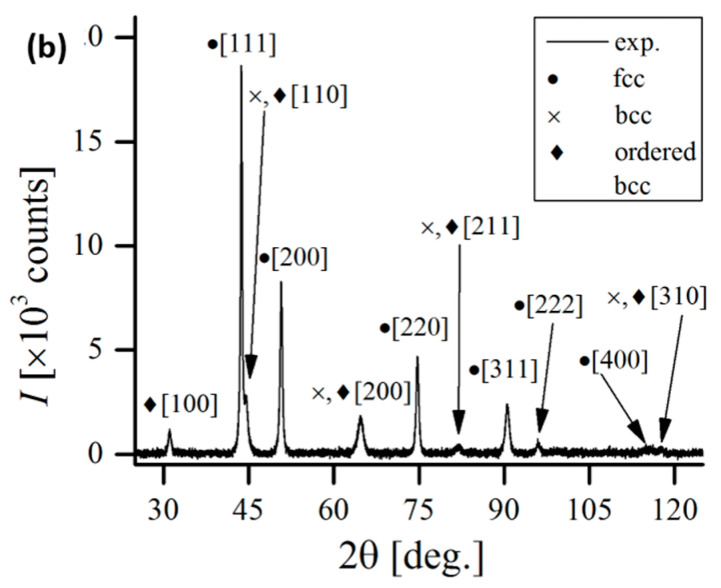
XRD patterns of analyzed HEAs: (**a**) Al_0.7_CoCrFeNi, (**b**) Al_0.7_CoCrFeNiV_0.5_.

XRD analysis confirmed the effect of vanadium on homogenizing the alloy. Figure 4 shows that in the case of the unmodified Al_0.7_CoCrFeNi alloy, two phases are present: fcc with a lattice constant of a_0_ = 3.5979 Å, and bcc with a lattice constant of a_0_ = 2.8879 Å. A comparison of the 110 peak for the bcc phase and the 111 peak for the fcc phase indicates that the volumetric fraction of the fcc phase is 84.9%, while the bcc phase accounts for 15.1%. These results align well with previous data obtained by the authors, published in studies [22], as well as in publications [19] focused on analyzing the effect of aluminum content on the structure and properties of the Al_x_CoCrFeNi alloy. These publications also highlighted the segregation of components between phases. The dendritic phase shows an increased concentration of aluminum and nickel, quantitatively corresponding to the fcc phase content, while the interdendritic regions exhibit a predominance of chromium and iron.

On the other hand, diffraction studies show that only a solid solution of alloying elements with a bcc structure is present in the alloy with 0.5 molar vanadium. Similarly, only large homogeneous grains without any segregation can be observed in the SEM images of Al_0.7_CoCrFeNiV_0.5_. It can therefore be concluded that vanadium stabilizes the bcc structure. These findings corroborate the research of Dong and his team presented in the paper [28].

### 3.2. Tribological Properties

**Figure 5:**
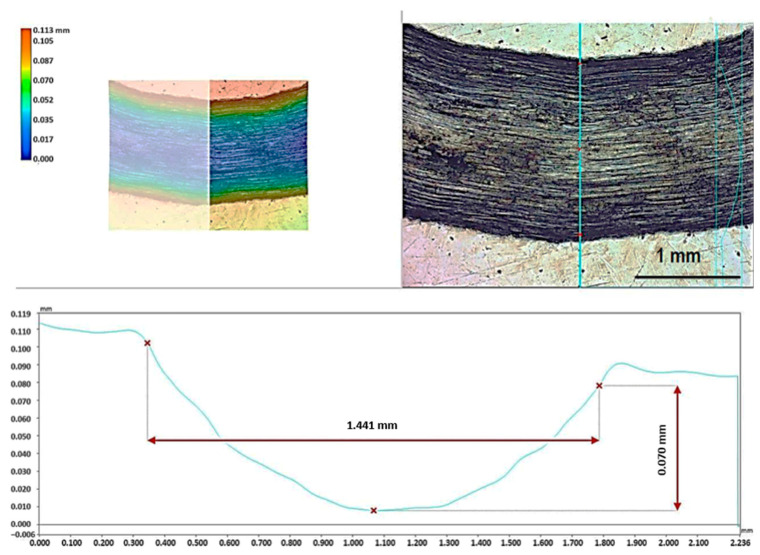
Measurement scheme of the linear wear of the Al_0.7_CoCrFeNi alloy in a friction path.

**Figure 6:**
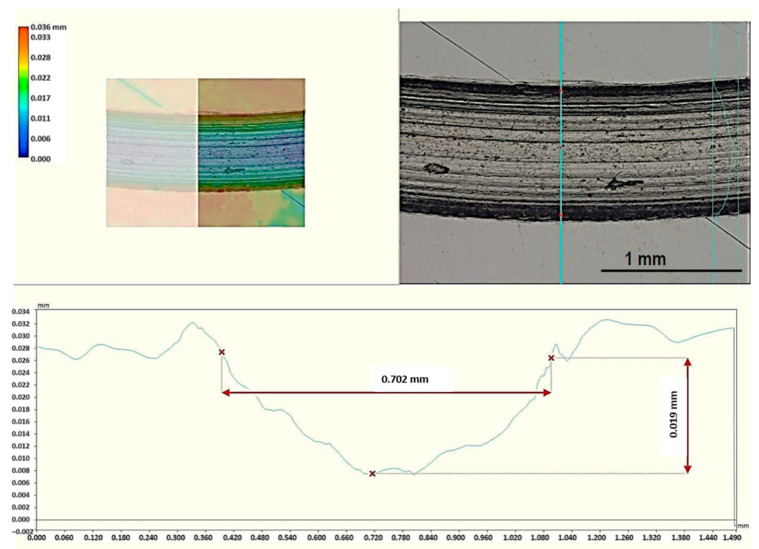
Measurement scheme of the linear wear of the Al_0.7_CoCrFeNiV_0.5_ alloy in a friction path.

Figure 5 and Figure 6 show illustrative results of the measurements of the wear rate on the friction paths for Al alloy and vanadium alloy, respectively. In the analyzed case, the tests were conducted under identical parameters for both materials. Therefore, linear wear, represented by the depth of the groove (friction track) in the radial section, was used for comparison.

Friction tests were conducted under identical conditions for both alloys. With a sliding speed of 0.1 m/s and a time of 1800 s, the friction path was 180 m, which, with a friction radius of 3 mm, results in approx. 9550 friction cycles. A comparison of the width of the friction paths in Figure 5 and Figure 6 shows that the friction trace is much narrower for the vanadium alloy. The groove depth was measured on a measuring microscope.

**Figure 7:**
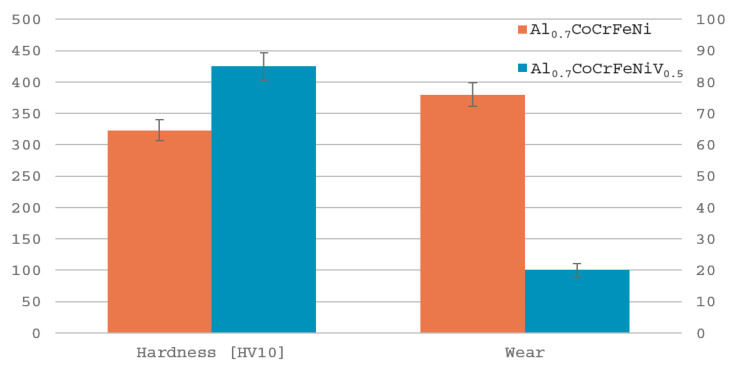
Comparison of hardness and depth of friction paths measurement of tested alloys.

The results of the tests are summarized in Figure 7 along with the materials’ hardness measurements. The addition of vanadium resulted in an increase in the hardness of the alloy by 33% and a significant decrease in friction wear value by 73%.

**Figure 8:**
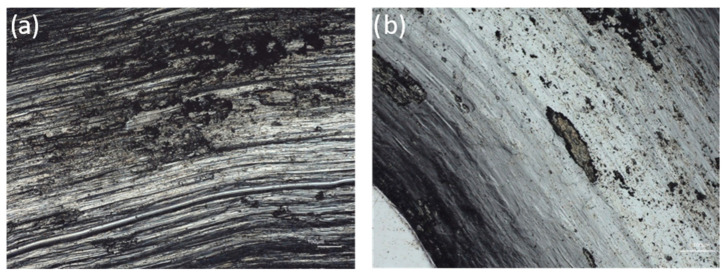
Wear tracks after friction on the surface of the following: (**a**) Al_0.7_CoCrFeNi, (**b**) Al_0.7_CoCrFeNiV_0.5_ alloy.

The friction traces of the studied materials are shown in Figure 8. It is noticeable that there is a completely different wear mechanism in the tested materials. On the microscopic picture of the Al_0.7_CoCrFeNi alloy surface (Figure 8a), practically all types of wear can be observed: abrasive wear with the formation of deep scratch marks, probably caused by hard wear products, adhesive wear with the detachment of material fragments, and the formation of a secondary layer together with wear products. However, in this case, the layer does not protect the material from further wear.

In contrast, the friction trace surface of the Al_0.7_CoCrFeNiV_0.5_ does not show the presence of a deposited secondary layer, only exposed substrate material (Figure 8b). This is evidenced by the clear grain boundaries of the base alloy. No signs of adhesive or fatigue wear are evident. Only occasional wear products of an oxide character are visible.

In order to understand the wear processes, detailed observations of the friction marks were carried out with a scanning electron microscope using an FIB to obtain cross sections of the surface layer after friction.

**Figure 9:**
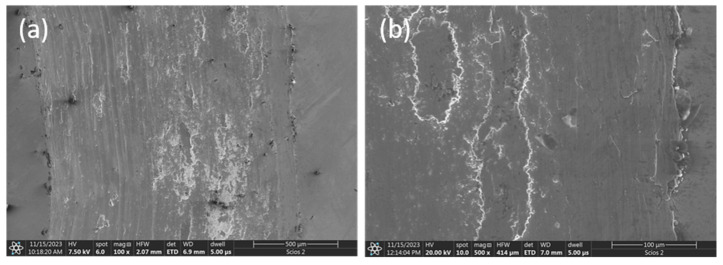
SEM micrographs of the worn surface of the Al_0.7_CoCrFeNi alloy: (**a**) magn. 100×, (**b**) magn. 500×.

**Figure 10:**
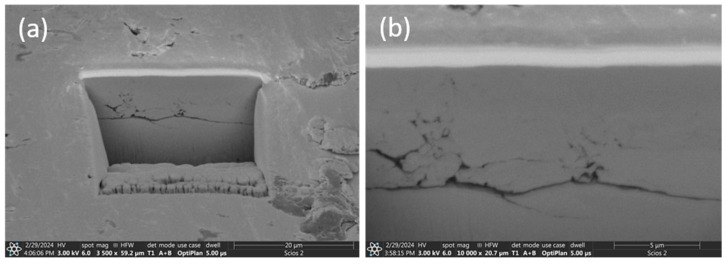
Cross section of the wear track of the Al_0.7_CoCrFeNi alloy: (**a**) Position of the cross-section on the friction track, magn. 3500×, (**b**) sectional view, magn. 10,000×.

An intense interaction with the substrate can be observed on the surface of the Al_0.7_CoCrFeNi base alloy (Figure 9a). The primary structure of the basic Al_0.7_CoCrFeNi alloy contains a high dendritic content of the soft fcc phase and a harder bcc phase in the interdendritic spaces (Figure 1). This structure is susceptible to fatigue wear during cyclic contact with the counterspecimen under load and to adhesive sizing. The material on the surface was abraded and many wear products were formed which, while remaining in the friction zone, continued to interact with the substrate. A cycle occurred in which wear products adhered to the substrate, then crumbled and were squeezed back into the surface, followed by new fragments being torn out. As a result, a surface layer, more than 6 μm thick, was formed over the entire surface of the friction trace, barely adhering to the substrate (Figure 10). The layer material was ductile, as evidenced by plastic extrusion at the margins of the trace. At the layer–substrate interface, traces of fatigue wear of the base material could be observed (Figure 10b), which indicates that the formed layer did not protect against further wear. Ploughing marks caused by hard wear particles as well as material detachment (Figure 8a) were visible on the surface of the layer. The detached material was subsequently transferred and smeared along the friction path (Figure 9b).

**Figure 11:**
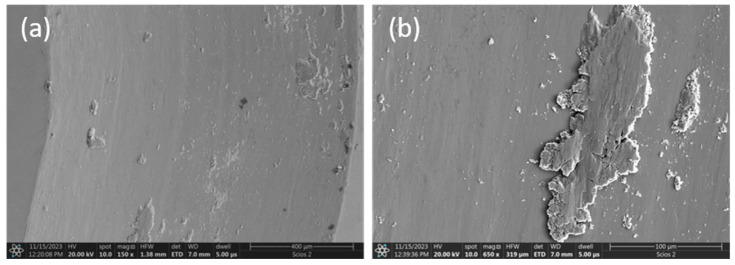
SEM micrographs of the worn surface of the Al_0.7_CoCrFeNiV_0.5_ alloy: (**a**) magn. 150×, (**b**) magn. 650×.

In contrast, the friction surface of the vanadium-added material presents a completely different situation. The friction surface is smooth, without any signs of adhesive wear or fatigue cracking. On the surface of the friction trace of the vanadium alloy sample, metal grains can be observed, characteristic of the microstructure of this alloy (Figure 8b). This means that the exposed surface of the material is visible. Figure 11 shows that only some sporadic wear products are visible on the surface, plastically deformed and smeared on the sliding truck.

**Figure 12:**
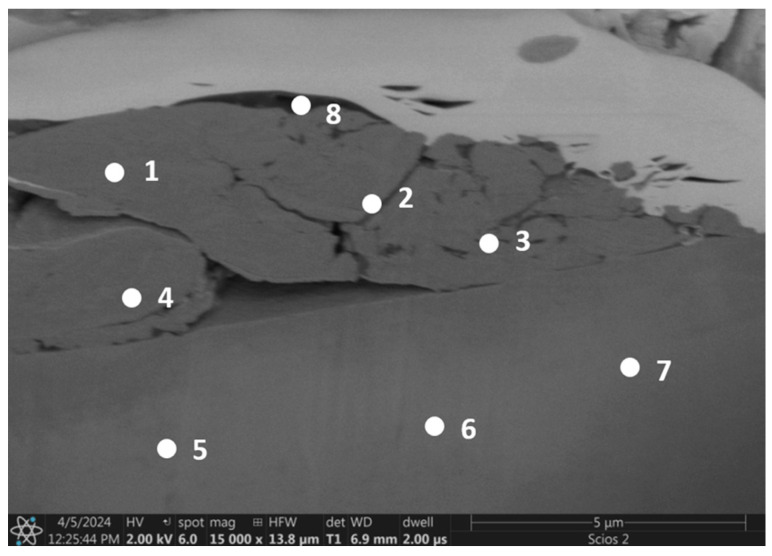
Cross section of the wear track of the Al_0.7_CoCrFeNiV_0.5_ alloy.

Table 1 shows the chemical composition of characteristic points on the cross section made on the surface of the alloy after friction (Figure 12). According to the chemical compositions, the wear products contain a certain amount of oxygen. The proportions of the components suggest that the wear debris was formed by the mixing of metallic material with oxides resulting from local surface oxidation. It is unlikely to contain material from the corundum counter body, as this would result in a higher aluminum content in measurement points 1–4 (Figure 12). However, the aluminum content is consistent between the wear debris and the base material. In contrast, the substrate material (points 5–7 in Figure 12) is characterized by an unchanged structure and chemical composition. The wear debris is loosely attached to the substrate, not contributing to adhesive wear. This observation is intriguing and warrants further investigation. It is possible that surface oxidation plays a protective role. Additionally, there are no deep grooves typically associated with abrasive wear. It can be inferred that the solid solution strengthening of the bcc alloy contributed to improved wear resistance during friction.

The presence of gallium and platinum residues is due to the FIB cutting technique used.

## 4. Conclusions

1. The addition of vanadium changed the microstructure of the alloy. The microstructure of the Al_0.7_CoCrFeNi alloy is two phase, consisting of a mixture of fcc and bcc phases with slightly different chemical compositions, mainly with different proportions of aluminum and chromium. On the other hand, the modification of the alloy with the addition of vanadium resulted in homogenization of the structure.
2. The addition of vanadium increased the hardness of the material by about 33%.
3. The proposed modification also increased the wear resistance of the alloy—the wear value decreased by 73%. Notably, the wear mechanism has also changed. In the case of the unmodified Al_0.7_CoCrFeNi alloy, the wear was of mixed adhesive–abrasive nature with the formation of a secondary layer. On the other hand, the Al_0.7_CoCrFeNiV_0.5_ alloy was dominated by abrasive wear with the presence of oxide wear products lifted from the friction zone.

## Figures and Tables

**Table 1:** Chemical composition of characteristic points on the cross section of wear product of the Al_0.7_CoCrFeNiV_0.5_ alloy (atomic%).

	O	Al	V	Cr	Fe	Co	Ni	Ga	Pt
1	9.20	7.36	9.90	11.95	20.00	20.06	20.67	0.67	0.20
2	8.59	7.24	10.11	12.30	20.54	20.47	20.40	0.27	0.08
3	9.60	6.92	10.03	12.16	20.12	20.08	19.73	0.28	0.00
4	8.24	6.40	10.80	12.79	20.63	20.87	19.86	0.30	0.10
5		6.63	12.40	14.16	22.74	22.55	21.27	0.24	
6		7.17	12.65	13.85	22.43	22.45	21.21	0.24	
7		6.62	11.76	14.01	22.90	23.05	21.43	0.23	
8	7.46	6.80	8.51	11.70	19.45	19.26	19.41	2.32	5.08

## Data Availability

The data presented in this study are available on request from the corresponding author.
